# Cbl-mediated K63-linked ubiquitination of JAK2 enhances JAK2 phosphorylation and signal transduction

**DOI:** 10.1038/s41598-017-04078-w

**Published:** 2017-07-04

**Authors:** Chun-Shan Liu, Hsin-Fang Yang-Yen, Ching-Shu Suen, Ming-Jing Hwang, Jeffrey Jong-Young Yen

**Affiliations:** 1 Graduate Institute of Life Sciences, National Defense Medical Center, Taipei, Taiwan, ROC; 2 Institute of Biomedical Sciences, Academia Sinica, Taipei, Taiwan, ROC; 3 Institute of Molecular Biology, Academia Sinica, Taipei, Taiwan, ROC

## Abstract

JAK2 activation is crucial for cytokine receptor signal transduction and leukemogenesis. However, the underlying processes that lead to full activation of JAK2 are unclear. Here, we report a positive role for ubiquitination of JAK2 during GM-CSF-induced activation. Upon GM-CSF stimulation, JAK2 ubiquitination is significantly enhanced through K63-linked poly-ubiquitination. Studies employing both knockout and overexpression of Cbl, an E3 ubiquitin ligase, led to the conclusion that Cbl specifically promotes JAK2 ubiquitination, and this was further confirmed *in vitro* using a Cbl ubiquitination assay. Moreover, following GM-CSF stimulation, the levels of phospho-JAK2 and -STAT5 and a STAT5 luciferase reporter assay were all reduced in Cbl knockout cells and this effect could be rescued by Cbl expression. Mechanistically, Cbl can interact with, and ubiquitinate JAK2 FERM and kinase domains via the Cbl TKB domain. Using lysine-to-arginine site-directed mutagenesis, K970 in the kinase domain of JAK2 was identified as the ubiquitination site important for promoting full JAK2 activation by Cbl via K63-conjugated poly-ubiquitination. Our study suggests that GM-CSF-induced JAK2 activation is enhanced by Cbl-mediated ubiquitination of JAK2. Targeting ubiquitination of JAK2 might offer a novel therapeutic strategy against JAK2-mediated disorders.

## Introduction

Janus kinase 2 (JAK2) is a member of the Janus kinase family, which belongs to the non-receptor tyrosine kinase superfamily. JAK2 is a key intracellular signaling molecule that couples type II cytokine receptors, including the receptors for growth hormone, erythropoietin, and granulocyte-macrophage colony-stimulating factor (GM-CSF), to downstream signaling pathways^1, 2^. Given the diversity of type II cytokine biology, JAK2 actively participates in many biological processes, including hematopoiesis and innate immune responses^3^. In 2005, a gain-of-function somatic JAK2 mutation, V617F, was identified to be highly prevalent in myeloproliferative disorders^4^. Patients with this gain-of-function mutation have frequently been identified in polycythemia vera (PV; 95%), essential thrombocythemia (ET; 20–40%), and primary myelofibrosis (PMF; 50%)^4–7^. These findings extend the importance of JAK2 dysregulation to include hematopoietic malignancies, in addition to the conventionally- recognized inflammatory and immunological disorders.

The architecture of JAK family proteins has been highly conserved through evolution. These proteins contain four conserved domains: FERM, SH2, JH2 pseudo-kinase, and JH1 kinase. The N-terminal FERM and SH2 domains interact with the cytoplasmic tails of cytokine receptors; this is an essential step in JAK kinase activation^8–10^. The JH1 domain is a *bona fide* protein tyrosine kinase that contains two tyrosine residues (Y1007, Y1008) in the conserved activation loop, which, in turn, control kinase conformation and activation when phosphorylated^11, 12^. The structure of the JH2 pseudo-kinase domain highly resembles a kinase domain but contains a shorter activation loop^13, 14^ and plays a negative auto-regulatory role on the kinase domain^15–18^.

Intensive research efforts have been focused on understanding the significance of phosphorylated tyrosine residues in JAK2, principally using site-directed mutagenesis of such amino acids. The current model for JAK activation is that, upon cytokine stimulation, JAK2 is phosphorylated at multiple sites, some of which are required for kinase activation, including Y1007/8, Y637, Y868, and Y972/966, possibly *via* promoting conformational changes. On the other hand, some of these sites are involved in down-regulation of JAK2 activation, such as Y317, Y570, Y913, and Y119, which may ensure tighter control of cytokine signaling^19, 20^.

In addition to phosphorylation, other post-translational modifications, including ubiquitination, have also been reported to control JAK2 stability and localization. Suppressor of cytokine signaling 1 (SOCS1) has been reported to inhibit cytokine-induced JAK2/STAT5 signaling through the ubiquitin-proteasome pathway^21–23^. The SOCS1 SH2 domain associates with JAK2 phospho-Y1007 in the activation loop, thereby blocking JAK2 catalytic activity. This association also leads to ubiquitin conjugation of JAK2, ultimately leading to its proteasomal degradation. Casitas B-lineage lymphoma (Cbl, also known as c-Cbl) is an E3 RING ubiquitin ligase that regulates the function of both receptor- and non-receptor tyrosine kinases, either through ubiquitination or *via* adaptor functions^24^. Cbl contains a tyrosine kinase-binding (TKB) domain at its N-terminus, followed by a linker region, a central zinc-binding C_3_HC_4_ RING finger motif, and a number of proline-rich motifs at the polypeptide C-terminus^24–26^.

Cbl is mainly expressed in hematopoietic cells^27, 28^. A germline Cbl mutation (Y371H) has been identified in 10–15% of juvenile myelomonocytic leukemia (JMML) patients. JMML is a disease characterized by overproduction of monocytic cells that are highly responsive to GM-CSF stimulation^29, 30^. Another Cbl mutation, C384R in the RING finger domain, has also been identified in myelodysplastic and myeloproliferative neoplasms^30^. Subsequent studies have revealed that homozygous *Cbl* mutations are present in most acquired uni-parental disomy myeloid malignancies, and that gain-of-function of mutations in *Cbl* are not associated with loss of the ubiquitin ligase activity, which is currently thought to play a tumor suppressing role^30, 31^. It is also reported that Cbl could serve as an adaptor for the GM-CSF receptor (GMR) β subunit and down-regulate levels of Src protein and its kinase activity, which, in turn, would limit GM-CSF-induced GMR activation^32, 33^. It was further suggested that, through their adaptor function, mutated Cbl proteins increase their association with Lyn kinase, as well as with the p85 regulatory subunit of PI3K, and thereby promote activation of Akt-dependent survival signals^34^.

In this study, we investigated the molecular mechanisms underlying Cbl-mediated JAK2 ubiquitination following GM-CSF induction. We demonstrate that GM-CSF stimulates the interaction between JAK2 and Cbl, which promotes JAK2 ubiquitination. Our results reveal that the K970 ubiquitination site on JAK2 is important for JAK2 phosphorylation, and for downstream signal transduction. Our studies identify a novel molecular mechanism that drives GM-CSF-induced JAK2 activation.

## Results

### GM-CSF induces predominantly K63-linked poly-ubiquitination of JAK2

**Figure 1:**
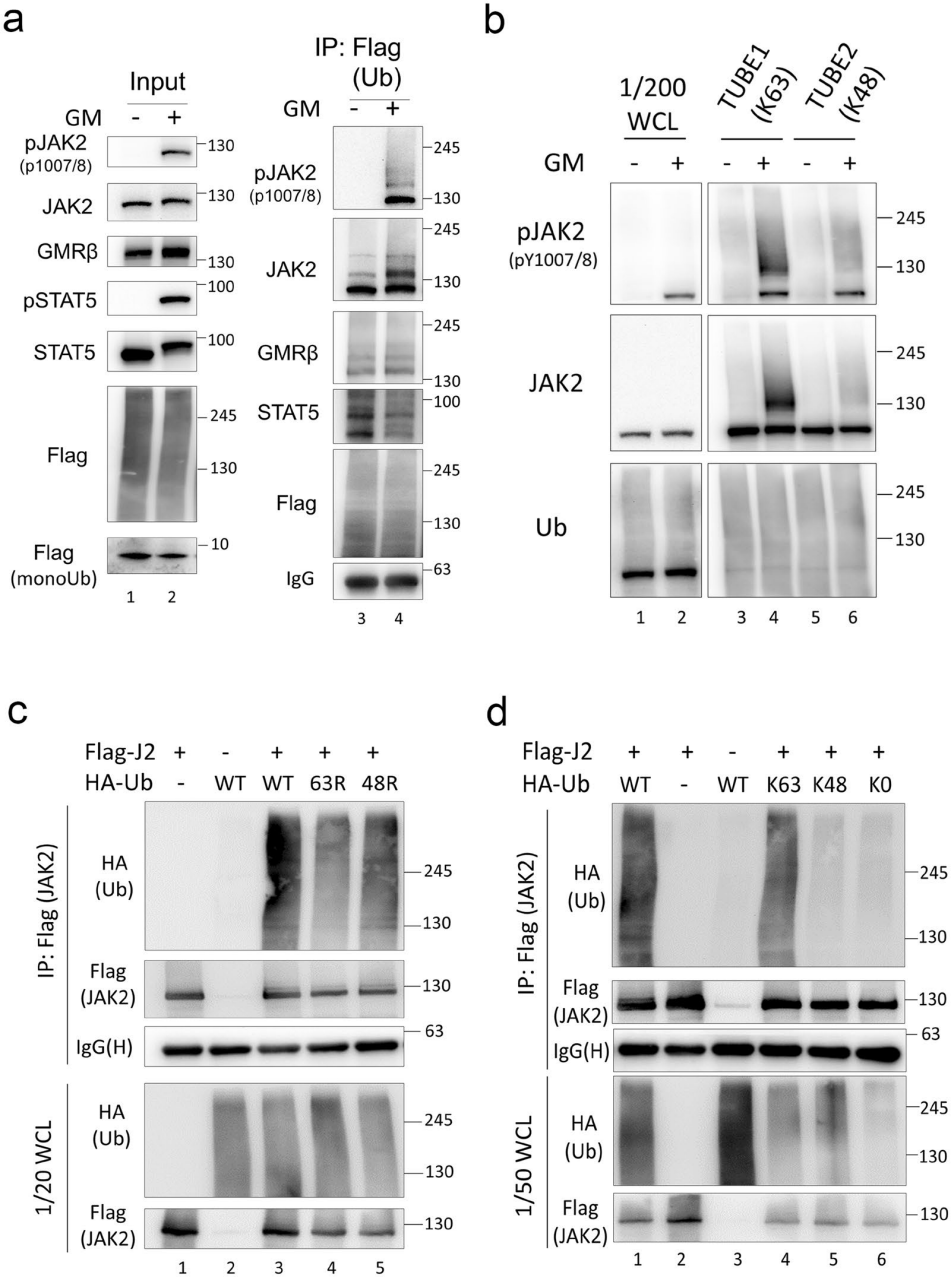
K63-linked poly-ubiquitin chain is predominant in ubiquitinated JAK2. (**a**) Ubiquitination of the GMRβ/JAK2/STAT5 signal axis during GM-CSF induced signaling. GMRαβ HeLa (GMR) cells were transfected with Flag-tagged ubiquitin. Transfected cells were serum-starved overnight, pretreated with MG132 (10 μM) for 4 h, and then stimulated with (+) or without (−) GM-CSF (1 ng/mL) for 10 min. Whole cell lysates (WCL) were immunoprecipitated (IPed) using an anti-Flag affinity resin, and Western blotting was performed for both the immunoprecipitates (IPs) and WCL using the indicated antibodies. (**b**) The K63-linkage is predominant in cytokine-induced JAK2 ubiquitination in TF1 cells. TF1 cells were serum-starved overnight, pretreated with MG132 (10 μM) for 4 h, and then stimulated with (+) or without (−) GM-CSF (GM) (1 ng/mL) for 10 min. Whole cell lysates were IPed using TUBE-1 or -2 beads and western blotting was performed for both the IPs and WCL (input control) using the indicated antibodies. (**c**) The ubiquitin K63R substitution greatly reduces JAK2 ubiquitination in 293 T cells. 293 T cells were co-transfected with Flag-tagged JAK2 and HA-tagged WT, K63R, or K48R ubiquitin mutants. Cells were processed and analysed as described above. (**d**) The K63-only mutant ubiquitin partially rescues ubiquitination of JAK2. 293 T cells were co-transfected with Flag-tagged JAK2 and HA-tagged WT or K63-only (K63), K48-only (K48), or the no-Lys (K0) mutant ubiquitin. Cells were treated and cell lysates were prepared and analysed as described in panel a. WCL: whole cell lysates. IgG(H) indicates IgG heavy chain from IP.

In our previous study, we demonstrated that GMRβ interacts with intersectin 2, and this interaction is required for ligand-induced JAK2 activation^35^. Intersectin interacts with Cbl to enhance EGFR ubiquitination and degradation following EGF stimulation^36^. Moreover, recent studies have indicated that Cbl is involved in GMR-mediated JAK2 activation^32, 33^, but the precise mechanism is not clear. To investigate the potential involvement of Cbl in the GMR/JAK2/STAT5 signaling axis, we sought to explore whether ubiquitination occurs during GM-CSF induced signaling. We transiently transfected Flag-tagged ubiquitin into HeLa cells stably expressing GMRα- and -β (GMR cells), and precipitated Flag-tagged ubiquitinated proteins. We observed that ubiquitination of JAK2 was clearly increased when JAK2 was phosphorylated upon GM-CSF stimulation, but was not elevated for GMRβ and STAT5 (Fig. 1a). To further distinguish the major type of poly-ubiquitin chain *in vivo*, we used a GM-CSF-dependent TF1 erythroleukemic cell line. Using Tandem Ubiquitin Binding Entity (TUBE)-1 and TUBE-2 beads (see Methods), which preferentially recognize K63- and K48-linked poly-ubiquitin, respectively, we observed that in the presence of proteasome inhibitor, MG132, ubiquitination of both endogenous phospho-JAK2 and JAK2 were significantly increased after GM-CSF stimulation in TUBE-1 immunoprecipitates (IPs) compared to TUBE-2 IPs (Fig. 1b). This suggested that K63-linked poly-ubiquitination is more predominant than K48-linked poly-ubiquitination, which was barely detectable. To further confirm this finding, mutant ubiquitins, containing lysine-to-arginine (KR) substitutions at either residue 48 (48 R) or 63 (63 R), were co-transfected with Flag-tagged JAK2 to repeat the experiment. As presented in Fig. 1c, in anti-Flag IPs, the 63 R mutation significantly reduced the ubiquitination level of Flag-JAK2, whereas the 48 R mutation had only a slight effect on ubiquitination. Moreover, when we co-expressed Flag-JAK2 with ubiquitin mutants containing either only the lysine 48 site (K48) or just the lysine 63 substitution (K64), it is apparent that the K63 mutant is capable of ubiquitinating JAK2 (Fig. 1d). Taking these data together, following GM-CSF stimulation, JAK2 is predominantly ubiquitinated *via* a K63-linked polyubiquitin chain.

### Cbl regulates JAK2 ubiquitination

**Figure 2:**
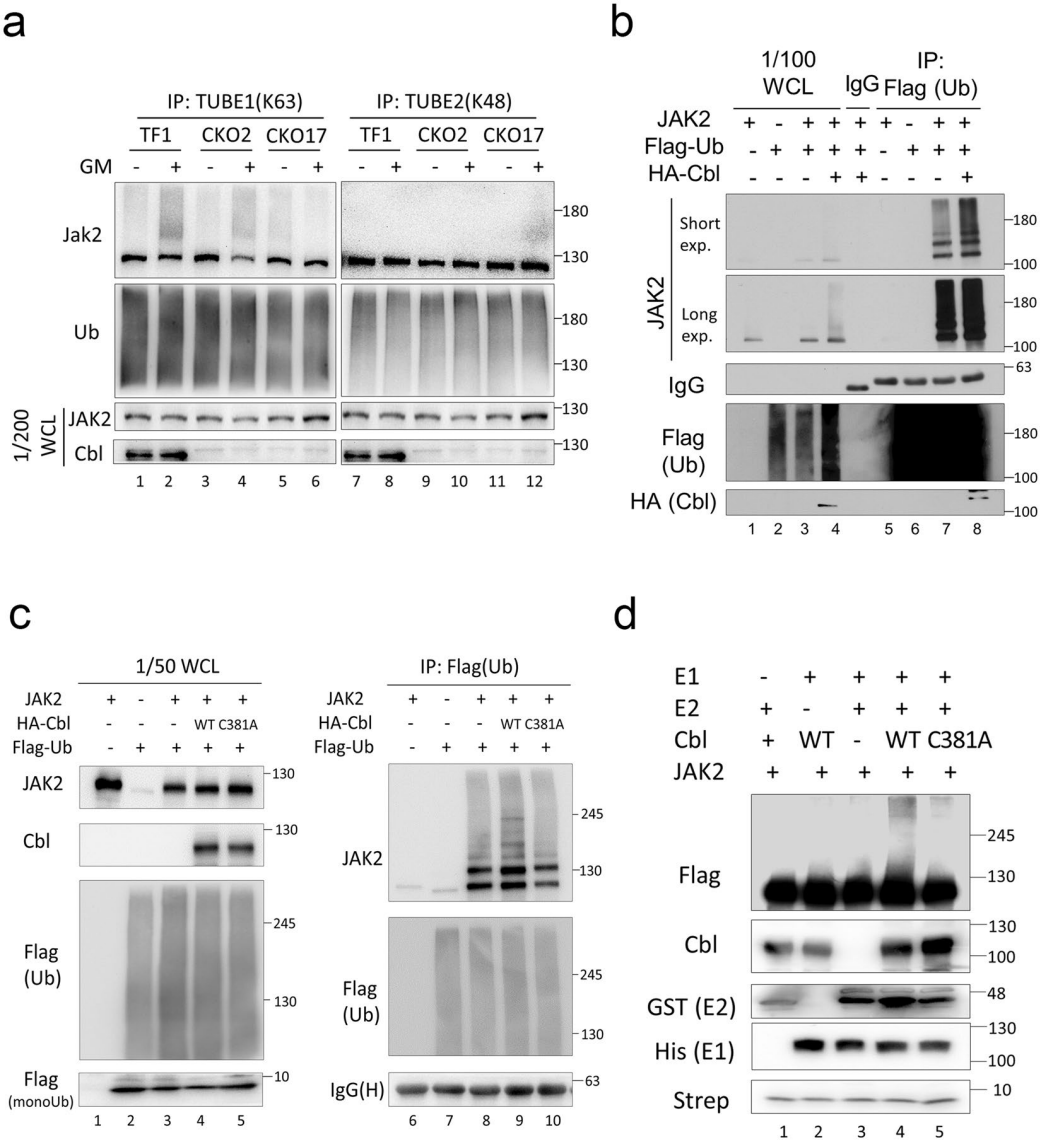
Cbl directly ubiquitinates JAK2 via the K63 linkage. (**a**) Cbl is involved in cytokine-triggered K63-linked ubiquitination of JAK2. After overnight serum starvation, parental TF1 cells (TF1) or Cbl knockout TF1 cells (CKO2 and CKO17) were processed and analysed as described in Fig. 1b. (**b**) Ectopic expression of Cbl enhances JAK2 ubiquitination. JAK2 and Flag-tagged ubiquitin were co-expressed in 293 T cells with or without HA-Cbl as described previously. WCL were prepared and analysed as described in Fig. 1c. A short and longer exposure of the JAK2 Western blot is shown for clarity. (**c**) Cbl rescues high molecular weight signals of JAK2 ubiquitination. JAK2 and Flag-tagged ubiquitin were co-expressed in Cbl knockout GMR HeLa (CKO) cells with or without HA-Cbl (wide type or C381A, a ligase-deficient mutant), then subjected to analysis as described in Fig. 1c. (**d**) JAK2 is directly ubiquitinated by Cbl in an *in vitro* assay. For the *in vitro* ubiquitination assay, Flag-tagged JAK2 and HA-tagged WT or C381A mutant Cbl were prepared as described in the Methods, and these were then incubated with recombinant E1, E2 (Ubc13), or both together, in the presence of biotin-labelled ubiquitin. After incubation, reactions were directly analysed by western blot with the indicated antibodies.

Next, to investigate whether Cbl is responsible for JAK2 ubiquitination, we generated two TF1 Cbl knockout cell lines (CKO2 and CKO17) by CRISPR/Cas9-based technology (see Methods) and used these to examine the effects on ubiquitination of endogenous JAK2 following GM-CSF stimulation. CKO2 and CKO17 cells were stimulated with GM-CSF and cell lysates were then IPed with TUBE-1 or TUBE-2 beads. GM-CSF-induced K63-linked JAK2 ubiquitination was reduced in CKO2 and CKO17 cells, compared to parental TF1 cells (Fig. 2a, lanes 2, 4 and 6). However, K48-linked JAK2 ubiquitination was undetectable in all cells (Fig. 2a). On the other hand, to address whether Cbl can promote JAK2 ubiquitination, we evaluated co-expression of JAK2 and Flag-tagged ubiquitin with or without HA-tagged Cbl. In 293 T cells, ubiquitination of JAK2 was detectable with endogenous Cbl, and this elevation was further increased when Cbl was overexpressed (Fig. 2b, compare lanes 7 and 8). When IPed with Flag-Ub, Cbl knockout HeLa cells gave a high JAK2 background signal even in the absence of Cbl (Fig. 2c, lane 8). However, expression of wild-type (WT) Ub significantly increased a ladder of signals in the higher molecular weight range (lane 9), and expression of C381A Cbl did not result in this ladder (lane 10), similar to that observed for the negative control (lane 8). Finally, to reinforce the notion that Cbl directly regulates JAK2 ubiquitination, an *in vitro* ubiquitination assay was employed. As shown in Fig. 2d, incubation of purified Flag-tagged JAK2 with recombinant E1, E2, and purified WT Cbl resulted in the appearance of a smeared signal of JAK2 by Western blot analysis (Fig 2d, lane 4). This smear of ubiquitinated JAK2 was not observed when purified JAK2 proteins were co-incubated with the Cbl C381A mutant (C381A) (Fig. 2d, lane 5). Although this *in vitro* ubiquitination assay was not fully optimized, it provided a proof-of-concept demonstration that Cbl directly promotes JAK2 ubiquitination without the cooperation of other factors, especially cytokine receptors.

### Cbl plays an important role in JAK2/STAT5 signaling activity in cells

**Figure 3:**
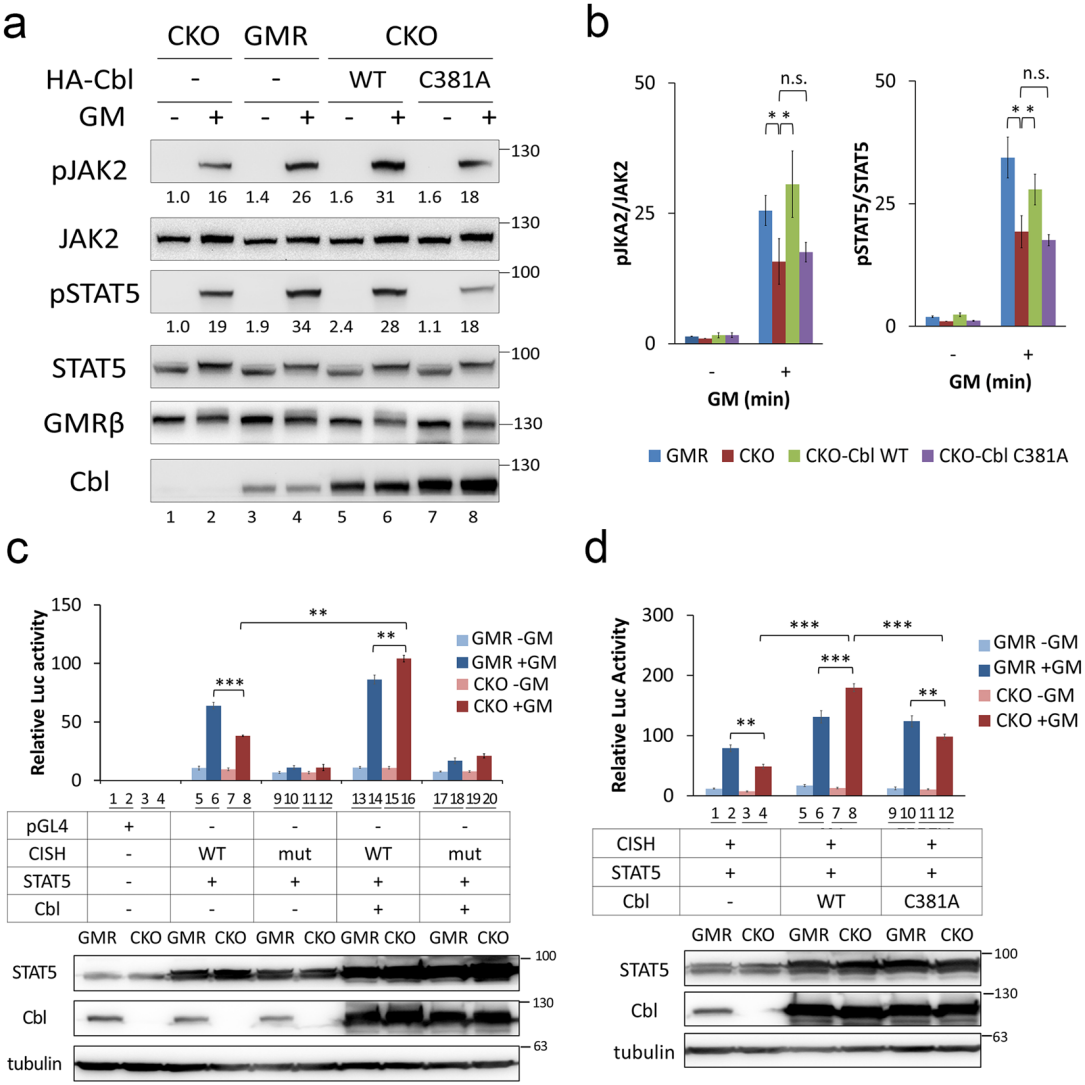
Cbl plays a positive role in GMRβ/JAK2/STAT5 signal axis in cells. (**a**,**b**) Cbl affects cytokine-induced JAK2 and STAT5 activation in GMRαβ HeLa (GMR) cells. (**a**) HA-tagged Cbl (wide type or C381A, a ligase-deficient mutant) were transiently expressed in Cbl knockout GMR cells (CKO). After serum starvation for 16 h, transfected cells, as well as the parental GMR cells, were stimulated with (+) or without (−) GM-CSF (GM) (1 ng/mL) for 10 min, and their WCL were analysed by Western blot using the indicated antibodies. (**b**) Quantification of the data from (**a**). (**c**) Cbl promotes STAT5-driven CISH reporter activity upon GM-CSF stimulation. CKO and parental GMR cells were transiently co-transfected with the indicated wild-type (WT) or mutant (mut) CISH reporter genes and STAT5a without (lanes 5–12) or with (lanes 13–20) Cbl rescue. Transfected cells were treated with (+), or without (−), GM-CSF (GM) (1 ng/mL) for 24 h prior to performing the luciferase assay. The lower panel illustrates the protein levels of endogenous and overexpressed STAT5 and Cbl, as well as tubulin for normalization, in the various transfected cells. (**d**) The ubiquitin ligase activity of Cbl is required for rescuing STAT-dependent reporter activity. Cells were transfected and analysed as described in (**c**), except that cells were rescued with WT Cbl (lanes 5–8) or a ubiquitin E3 ligase dead mutant (C381A Cbl) (lanes 9–12). In (**c**) and (**d**), the relative luciferase values in the upper panel are presented as mean ± SEM. All experiments were repeated three times. Statistical significance (*P < .05, **P < 0.01 and ***P < 0.001) was determined by t-test.

In line with the observation that JAK2 ubiquitination predominantly involves K63, we did not detect significant changes in JAK2 protein levels, with or without cytokine stimulation or treatment with proteasome inhibitor. To explore whether K63-linked ubiquitination of JAK2 is involved in JAK2 signaling, rather than in protein stability, we first investigated whether GM-CSF-stimulated signaling is affected in CKO cells. As shown in Fig. 3a, when stimulated with GM-CSF, both phospho-JAK2 Y1007/8 and phospho-STAT5 levels were significantly reduced in CKO cells compared to the parental GMR cells (comparison of lanes 2 and 4), and these reductions could be rescued by transient expression of wild type Cbl but not by the C381A mutant (comparison of lanes 2, 6, and 8, and Fig. 3b). To further demonstrate that the effect on JAK2/STAT5 phosphorylation is functionally relevant, we transfected a reporter plasmid that encodes a luciferase gene driven by a STAT5 responsive promoter from *cytokine-inducible SH2-containing protein* (*CISH)* gene (pGL4-CISH)^37^, into GMRαβ-expressing CKO HeLa cells to monitor the function of the JAK2/STAT signaling pathway. As shown in Fig. 3c, luciferase activity was significantly induced by GM-CSF stimulation in the presence of a reporter with a WT promoter sequence (lanes 5–8, lanes 13–16), but was not up-regulated by a reporter harboring a mutated STAT5 binding site (CISHmut) (lanes 9–12, lanes 17–20). Moreover, ligand-induced reporter activities were significantly affected by cellular levels of Cbl; the reporter activity was higher in control cells than in CKO cells (comparison of lanes 6 and 8), whereas reporter activity was higher in Cbl-transfected cells than in parental cells (comparison of lanes 14 and 16 to lanes 6 and 8). To further explore the contribution of the ubiquitin E3 ligase activity of Cbl, we replaced Cbl with a ligase-dead Cbl mutant, Cbl C381A, and performed the same experiment described above. As shown in Fig. 3d, although there was no obvious effect of the mutant on GM-CSF signaling in parental cells (comparison of lanes 5 and 6 and 9 and 10), there was a significant reduction in reporter activity observed when C381A Cbl was introduced into CKO cells (comparison of lanes 7 and 8 and 11 and 12), suggesting that the enzymatic function of Cbl plays a positive role in signal transduction along the JAK2/STAT5 axis. Intriguingly, there was also a significant difference in reporter activity when Cbl-C381A-expressing CKO cells were compared to vector-transfected CKO cells (lanes 12 and 4), suggesting that an additional effect, such as an adaptor function of Cbl, might also contribute in this functional assay system.

### Interaction of Cbl with JAK2 is enhanced by GM-CSF

**Figure 4:**
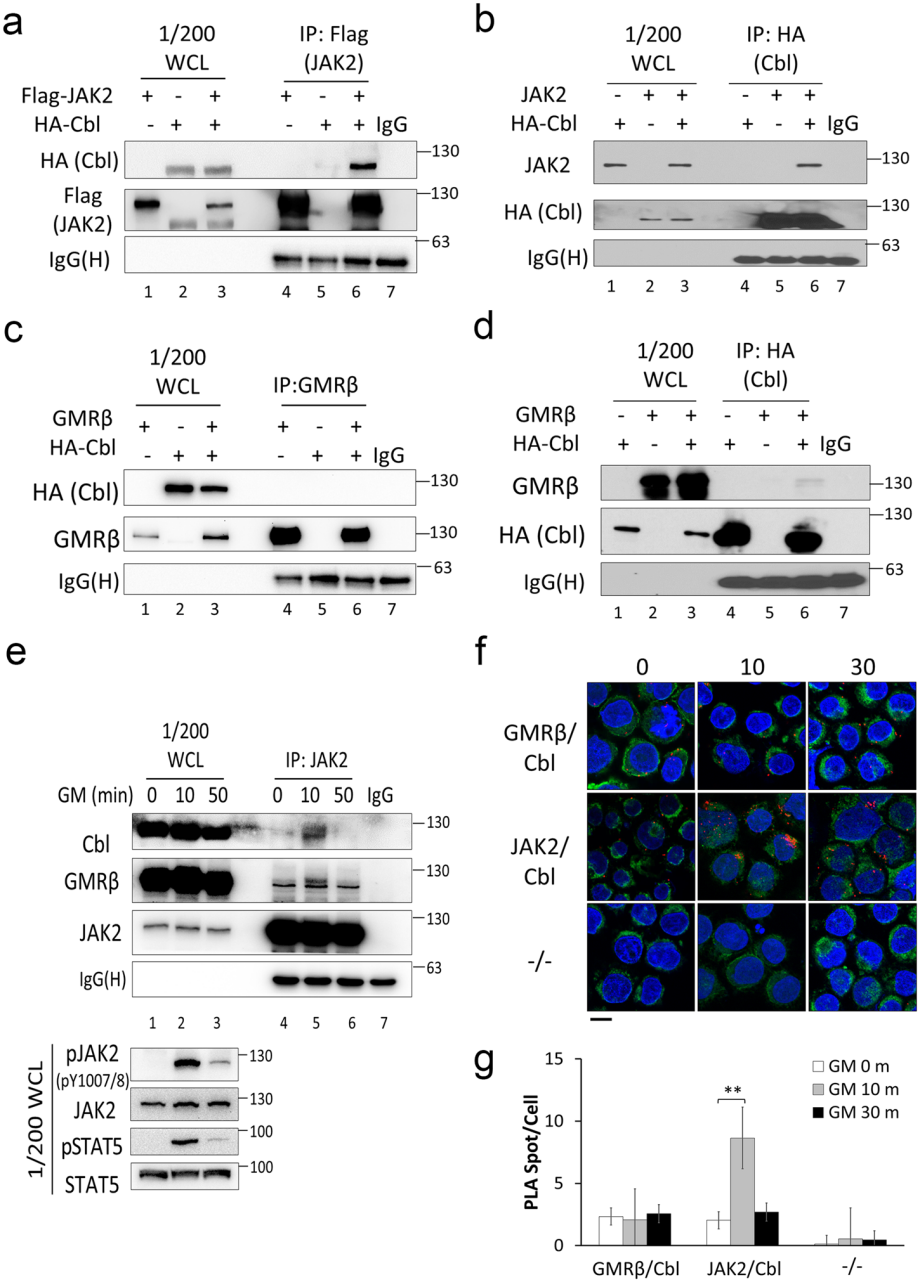
Cbl interacts with JAK2 during GM-CSF stimulation. (**a**,**b**) The interaction between JAK2 and Cbl. 293 T cells were co-transfected with combinations of plasmids encoding HA-tagged Cbl and Flag-tagged (**a**) or non-tagged JAK2 (**b**), and their WCL were IPed using an anti-Flag affinity gel (**a**) or an anti-HA affinity resin (**b**). The IPs, as well as their respective WCL (input control), were analysed by Western blot using the indicated antibodies. (**c**,**d**) Analysis of the interaction between GMRβ and Cbl. 293 T cells were co-transfected with combinations of plasmids encoding HA-tagged Cbl and un-tagged GMRβ, and WCL were IPed with (**c**) anti-GMRβ-protein G beads or (**d**) an anti-HA affinity resin. Western blot analysis was performed as described above. (**e**) The interaction between endogenous JAK2 and Cbl is cytokine-dependent. After serum starvation overnight, TF1 cells were stimulated with or without GM-CSF (GM) (1 ng/mL) for 10 or 50 min, and WCL were IPed with an anti-JAK2 antibody. Cells were analysed as described above. (**f**,**g**) Cytokine-induced endogenous protein association was detected using Duolink® *in situ* PLA. After serum starvation overnight, TF1 cells were treated with or without GM-CSF for 10 to 30 min. Cells were fixed onto glass slides and then co-stained with a rabbit anti-Cbl antibody and either mouse anti-GMRβ (top row) or mouse anti-JAK2 antibodies (middle row), or no primary antibody control (bottom row). The PLA reaction products (PLA spots, red) were detected by confocal microscopy. Nuclei were stained with DAPI (blue), and the plasma membrane was stained using CellMask™ plasma membrane stain (green). The scale bar represents 10 μm. (**g**) Quantitation of data in (**f**) obtained from at least 500 cells per condition. Data are presented as mean ± SEM from two independent experiments. Statistical significance (**P < 0.01) was determined by t-test. WCL: whole cell lysates. IgG(H): IgG heavy chain.

We further explored the question of whether Cbl can interact with GMR or JAK2 *in vivo*. HA-tagged Cbl, GMRβ, or Flag-tagged JAK2 were co-expressed in 293 T cells and their respective interactions were analysed by IP with anti-Flag, anti-HA, or anti-GMRβ antibodies. As shown in Fig. 4a,b, Cbl consistently interacted with JAK2, as revealed by either anti-Flag (Fig. 4a) or anti-HA (Fig. 4b) IPs. However, Cbl only weakly interacted with GMRβ (Fig. 4c,d). The interaction between endogenous JAK2 and Cbl was also examined in TF1 cells. Following IP with an anti-JAK2 antibody, we found that Cbl and JAK2 did undergo interaction; and this increased after 10 min of GM-CSF treatment, but was no longer evident after 50 min (Fig. 4e). A proximity ligation assay (PLA) was also employed to monitor the association between Cbl and JAK2 or GMRβ *in situ* (see Methods). As presented in Fig. 4f,g, both JAK2 and GMRβ exhibit only a low level of interaction with Cbl before stimulation. Upon GM-CSF stimulation, the number of JAK2-Cbl interaction spots increased significantly from 2 to 10 interaction-spots per cell at the 10 min time point and then returned to the basal level by 50 min (Fig. 4f,g); no changes in GMRβ-Cbl interaction occurred following GM-CSF stimulation. Taken together, both co-IP and PLA studies suggest that Cbl specifically interacts with the JAK2 kinase following GM-CSF stimulation in hematopoietic cells.

### FERM and kinase domains of JAK2 are bound and ubiquitinated by Cbl

**Figure 5:**
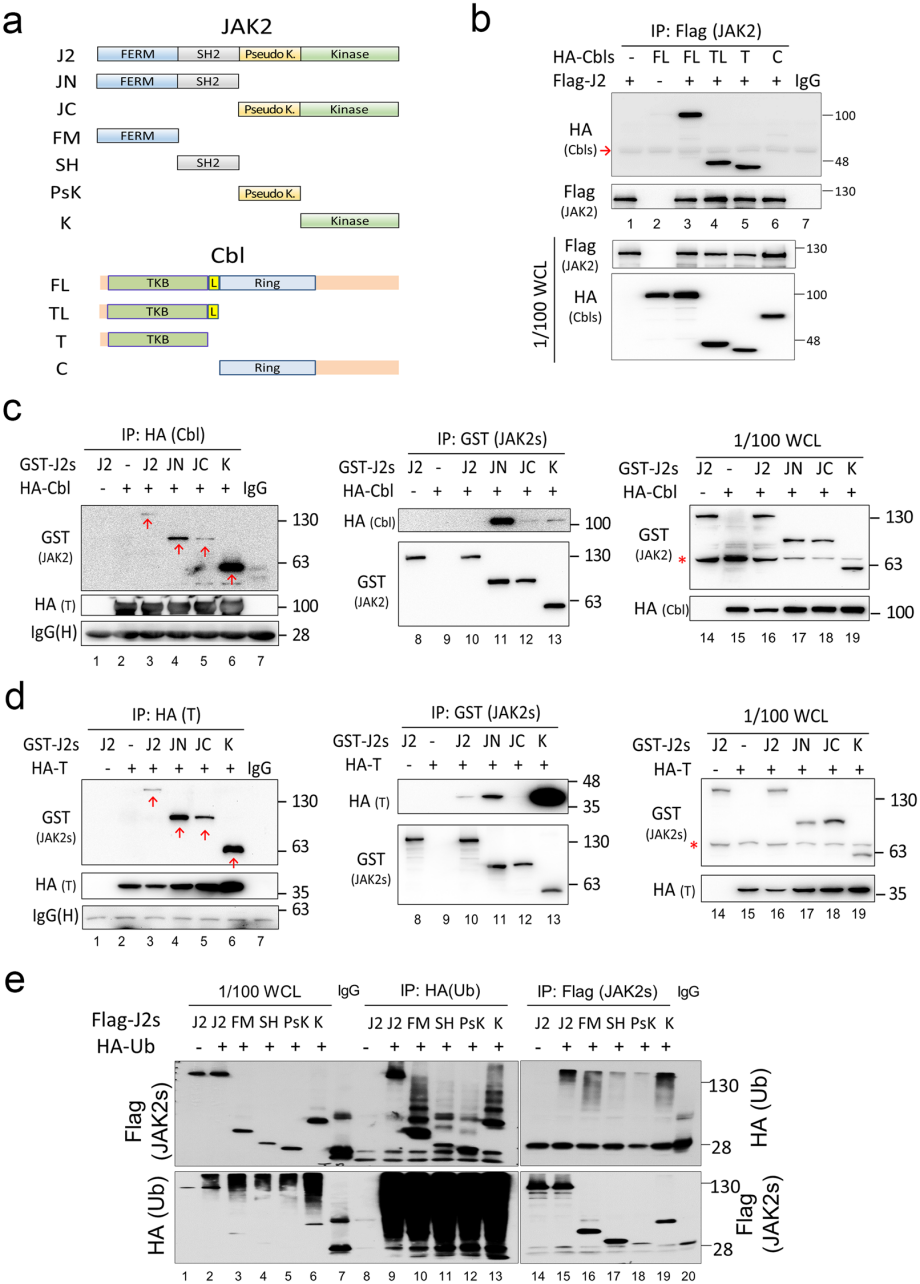
FERM and kinase domains of JAK2 are preferentially bound and ubiquitinated by Cbl. (**a**) Schematic representations of the full-length and sub-domains of JAK2 and Cbl that were used in the following experiments. (**b**) Interaction between full-length JAK2 and Cbl domains. 293 T cells were co-transfected with full-length (FL) Flag-tagged JAK2 and the various HA-tagged Cbl domains. WCL were IPed with an anti-Flag affinity gel, and the IPs as well as the WCL (input control) were analysed by Western blotting with the indicated antibodies. Red arrow indicates the IgG heavy chain from IP. (**c**,**d**) Interaction between Cbl and domains of JAK2. 293 T cells were co-transfected with the indicated GST-tagged domains of JAK2 and/or HA-tagged full length Cbl (FL) (panel c) or the TKB domain of Cbl (T) (panel d). Cells were then analysed as described above. The right hand column displays Western blots of the respective WCLs (input control). Red arrow indicates the different JAK2 fragments. Red asterisk marks a non-specific band. (**e**) Ubiquitination of JAK2 domains. 293 T cells were co-transfected with HA-tagged ubiquitin and various Flag-tagged JAK2 domains shown in (**a**). WCL were IPed and analysed as described above. IgG(H): IgG heavy chain. WCL: whole cell lysates.

We further searched for the domain(s) that mediate interaction between Cbl and JAK2. Along with full-length HA-tagged Cbl (FL), we generated three truncation mutants: the TKB domain plus linker region (TL), the TKB domain (T) only, and the C-terminal region (C) only and co-expressed these along with full-length Flag-tagged JAK2 (Fig. 5a). Following anti-Flag IP of full-length JAK2, we observed that FL-Cbl, TL-Cbl, and T-Cbl, but not C-Cbl, were co-IPed (Fig. 5b). These results suggest that Cbl interacts with JAK2 mainly *via* the TKB domain of Cbl. Conversely, HA-tagged full-length Cbl was co-expressed with various GST-tagged truncated JAK2 mutants: the N-terminal region containing the FERM and SH2 domains (JN), the C-terminal region containing the pseudo-kinase and kinase domains (JC), and the kinase domain only (K) (Fig. 5a,c). When cell lysates were IPed with an anti-HA antibody (to capture Cbl), all JAK2 truncated mutants were co-IPed with Cbl (Fig. 5c, left panel). However, the interaction of full-length J2 and JC with Cbl was both greatly reduced in comparison to JN and K (Fig. 5c, comparison of lanes 3 and 5 with lanes 4 and 6), and this was also observed in the reciprocal anti-GST IP, where J2 and JC were poorly precipitated along with HA-Cbl (Fig. 5c, middle panel, lanes 10 and 12), suggesting that full-length JAK2 and JC exhibited reduced binding interactions with Cbl. Since Cbl mainly binds JAK2 through the TKB domain (Fig. 5b), these co-IP studies were repeated using the TKB domain of Cbl. As shown in Fig. 5d, as was previously noted for FL Cbl, the TKB domain of Cbl was able to interact with all JAK2 subdomains, but with a preference for the JN and K domains; this was observed regardless of which binding partner was IPed, being either anti-HA (for Cbl) (Fig. 5d, left hand panel) or anti-GST (for JAK2) (Fig. 5d, middle panel) antibodies. These preferences could be explained by the influence of the highly folded structure of full-length JAK2 and the known interaction between the pseudo-kinase and kinase domains^16, 17^.

We next examined which domain(s) of JAK2 are conjugated by ubiquitin. To address this issue, we co-expressed HA-tagged ubiquitin along with different Flag-tagged JAK2 domains (FERM, SH2, pseudo-kinase and kinase, Fig. 5a). As shown in Fig. 5e, when ubiquitin was IPed with the anti HA antibody, the anti-flag Western blot revealed an evident ladder for the FERM and kinase domains (lanes 10 and 13), but not for the SH2 and pseudo-kinase domains (lanes 11 and 12). When the JAK2 domains were precipitated with the anti-Flag antibody, the anti-HA Western blot again showed a smear for the FERM and kinase domains (lanes 16 and 19) but not for the SH2 and pseudo-kinase domains (lanes 17 and 18). Full-length JAK2 also presented a smear (lane 15), which is more obvious than that seen in the anti-Flag Western blot (lane 9). Therefore, the major ubiquitination sites of JAK2 are in the FERM and kinase domains, which are also the domains preferentially bound by Cbl.

### Ubiquitination of the JAK2 kinase domain plays an important role in JAK/STAT signaling

**Figure 6:**
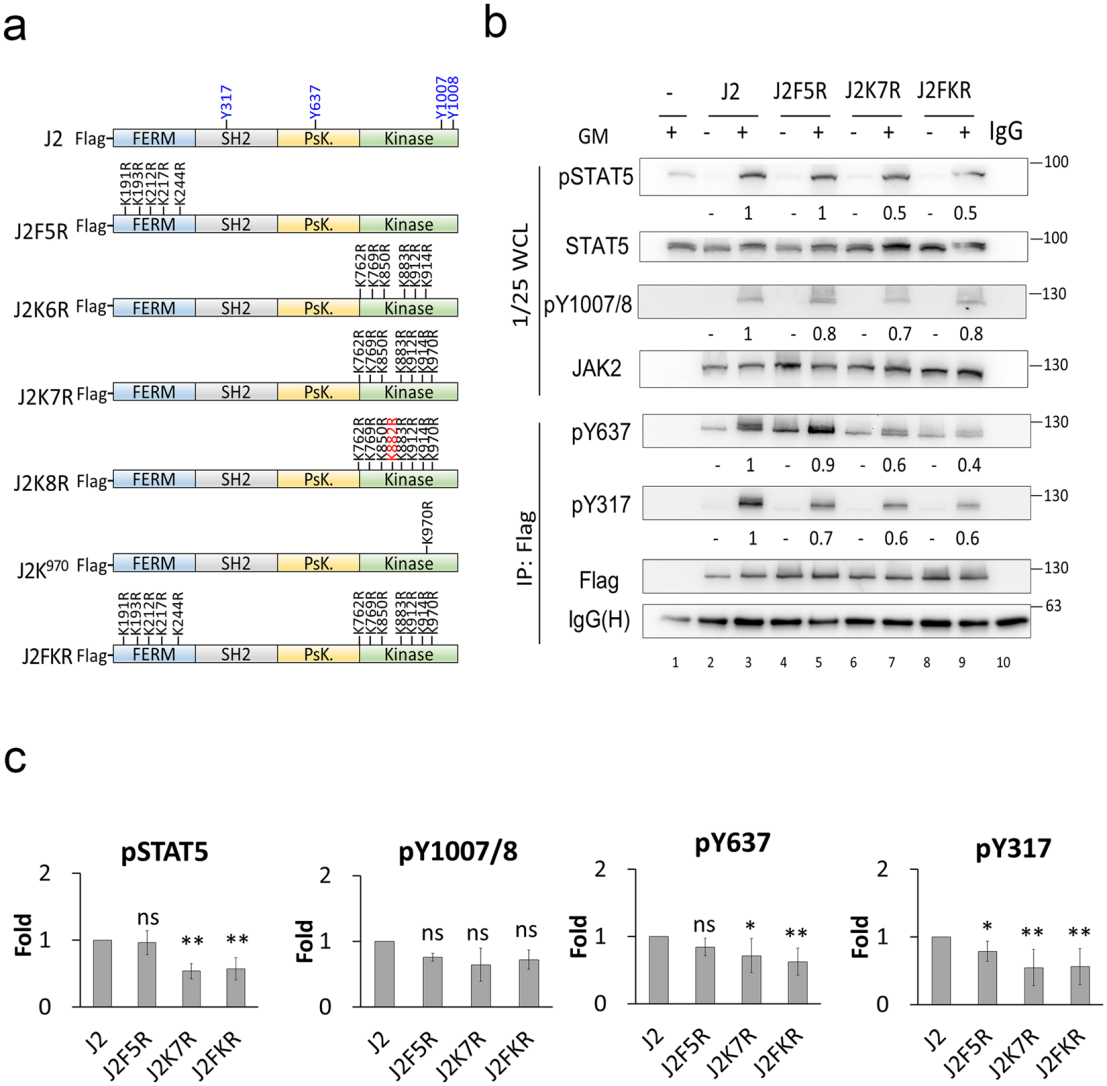
Ubiquitination of the kinase domain plays an important role in JAK2/STAT5 signal axis. (**a**) Schematic representation of KR mutants in the FERM and/or kinase domain of JAK2 that were used in the following experiments. Several important tyrosine residues that are phosphorylated during JAK2 activation are indicated in blue in the top panel. The kinase-dead JAK2 mutant (K882R) is marked in red. (**b**,**c**) Ubiquitination of the kinase domain is involved in cytokine-triggered JAK/STAT signaling. JKO cells were transfected with the indicated Flag-tagged JAK2 KR mutants (as shown in Fig. 6a), and, after overnight serum starvation, transfected cells were treated (+) or not treated (−) with GM-CSF (GM) (1 ng/mL) for 10 min. The WCLs were either analysed directly by Western blotting for levels of phospho-STAT5, total STAT5, phospho-JAK2 (Y1007/1008), and total JAK2, or were subjected to IP with an anti-Flag affinity gel and the IPs were probed for JAK2 phosphorylation at Y637 and Y317, as well as for Flag and IgG (b, lower panel). The phosphorylation levels on JAK2 and STAT5 shown in (**b**) were quantified and data are presented as means ± SEM from five independent experiments relative to WT JAK2 control, which was set as “1” (**c**). Statistical significance (*P < 0.05 and **P < 0.01) was determined by t-test. IgG(H): IgG heavy chain. WCL: whole cell lysates.

To explore which ubiquitination site in JAK2 is important in the JAK/STAT signaling pathway, we first employed a proteomic approach to identify the overall ubiquitination sites on JAK2. Based on the results from LC-MS/MS analyses, numerous ubiquitin binding sites in JAK2 proteins purified from JAK2-transfected 293 T cells were identified (data not shown). Because the JAK2 FERM and kinase domains are two main targets of ubiquitination, we focused on analysing the importance of five ubiquitination sites in FERM and seven in the kinase domain (excluding the catalytic lysine residue K882) that were identified at least twice in three independent experiments (for a schematic of the sites identified see Fig. 6a). To cover subtle biological effects, we also analysed the phosphorylation levels of Y317, a feedback-inhibitory site, and Y637, a positive regulatory site, in addition to the Y1007/8 sites to reflect the JAK2 activation status. We created mutants in the Flag-tagged full-length JAK2, which converted all lysine residues in either the FERM domain (J2F5R) or/and the kinase domain (J2K7R or J2FKR respectively) of JAK2 to arginine residues (Fig. 6a), and then analysed JAK2 and STAT5 tyrosine phosphorylation status upon GM-CSF stimulation in JAK2 knockout GMR HeLa (JKO) cells. As shown in Fig. 6b,c, although there was a slight reduction in the phosphorylation of Y317 (0.7-fold relative to wild type JAK2) when all five lysine residues were changed to arginine in the FERM domain, the levels of phosphor-Y637, -Y1007/8 and -STAT5 did not reach any significant changes (Fig. 6c). The phosphorylation levels of these sites in JAK2 exhibited a much more significant decrease when the kinase domain alone (J2K7R) or the FERM/kinase domain together (J2FK/R) were mutated (reduced to 0.6 and 0.4). These results suggested that ubiquitination of the kinase domain may play a more important role in regulating JAK2 activation.

### JAK2 K970 is important for JAK2 activation and downstream signaling

**Figure 7:**
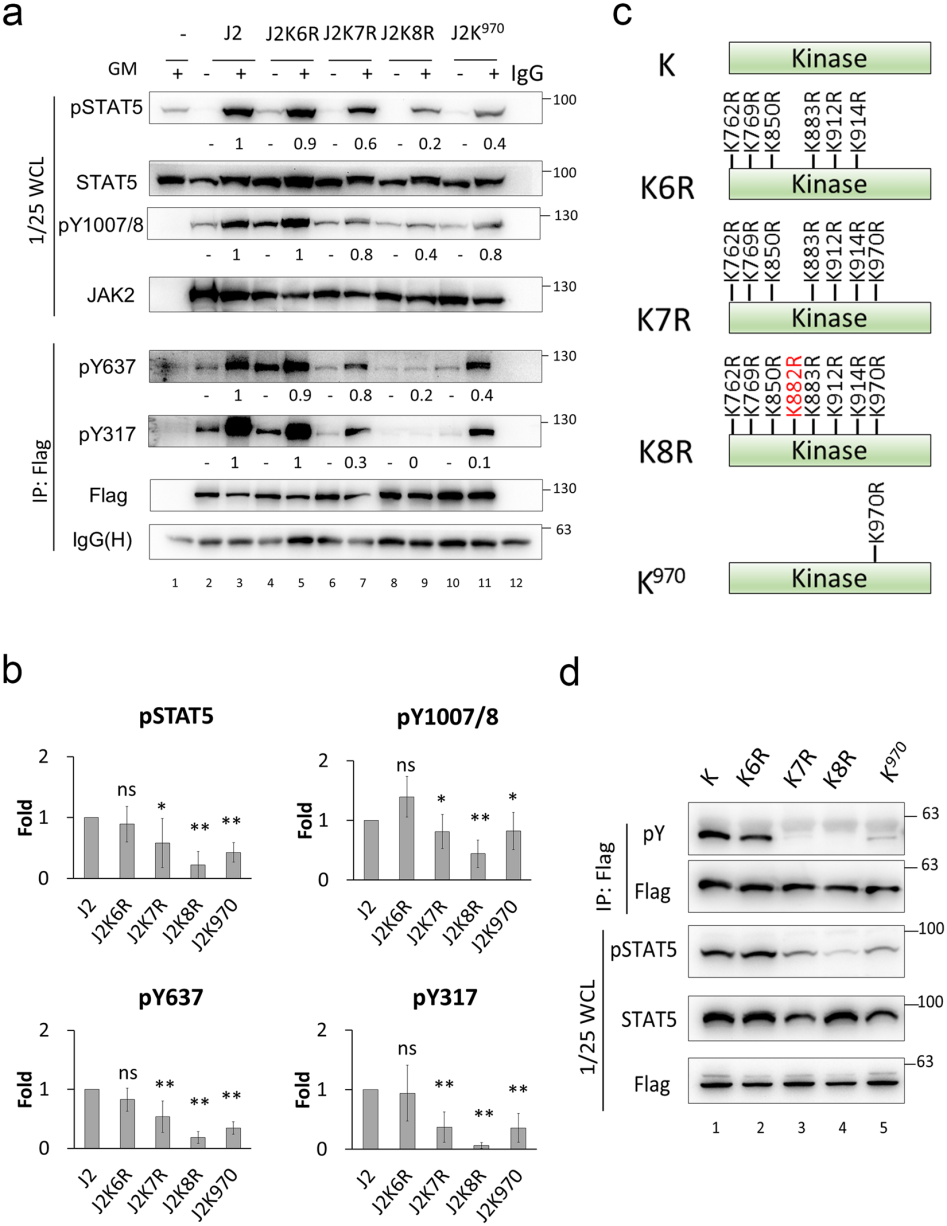
K970 in the JAK2 kinase domain is important for JAK2 activation and downstream signaling. (**a**,**b**) KR mutation at JAK2 K970 reduces GM-CSF-triggered JAK/STAT signaling. JKO cells were transfected with the indicated Flag-tagged JAK2 KR constructs and after overnight serum starvation the transfected cells were treated and analysed as described in Fig. 6. The phosphorylation levels on JAK2 and STAT5 shown in (**a**) were quantified and data are presented as means ± SEM from five independent experiments relative to WT JAK2 control, which was set as “1” (**b**). Statistical significance (*P < 0.05 and **P < 0.01) was determined by t-test. IgG(H): IgG heavy chain. (**c**) Schematic representations of KR mutants in the JAK2 kinase domain that were used in the *in vivo* kinase assay. The KR mutation at K882 (marked in red) in the JAK2 kinase domain is a kinase-dead mutant that serves as a negative control. (**d**) Auto-phosphorylation ability of JAK2 kinase domain is diminished by KR mutation at K970. 293 T cells were transfected with the indicated Flag-tagged JAK2 kinase domain KR mutants and WCLs were either IPed with an anti-Flag affinity gel and analysed by Western blotting using an anti-pY antibody, or were analysed for the levels of total and phospho-STAT5. Total Flag-tagged protein levels were used to normalize JAK2 expression.

Next, to identify which specific lysine residue(s) in the kinase domain is important for JAK2 activation, we generated a series of lysine-to-arginine (KR) mutants containing eight, seven, and six mutations (i.e. J2K8R, J2K7R, and J2K6R) or a specific K^970^-only mutation (J2K^970^, as described in Fig. 6a), and transfected these into JKO cells for signaling analysis. J2K8R is a kinase-dead mutant, wherein the catalytic lysine 882 is converted to inactive arginine, and was used as a negative control (lane 9). The J2K7R mutation restored the K^882^ ATP-binding site, and the J2K6R substitution restored the additional site, K^970^. As shown in Fig. 7a,b, the phosphorylation levels of Y317 were clearly decreased in the J2K7R and J2K^970^ mutants, and phosphorylation was restored in J2K6R (0.3 and 0.1 vs. 1). Similar results were also seen for phosphorylation levels at Y637 and Y1007/1008 in J2K7R and J2K^970^. To further demonstrate that lysine 970 is important for JAK2 kinase activity, an *in vivo* auto-phosphorylation assay using overexpression of the kinase domain was employed in 293 T cells. We expressed various Flag-tagged JAK2 kinase domain-only mutants (K6R, K7R, K8R, and K^970^; Fig. 7c) in 293 T cells and captured these by an anti-Flag M2 affinity gel and subjected them to Western blot analysis using anti-phospho-Tyr antibody. Western blotting revealed that K8R completely lost tyrosine auto-phosphorylation activity, and that K7R and K^970^ retained only a very low level of such activity (Fig. 7d). Interestingly, STAT5 phosphorylation also decreased in K7R- and K^970^-transfected cells, and STAT5 phosphorylation was rescued when lysine 970 remained (K6R) (Fig. 7d, lane 2). These results strongly supported that ubiquitination of lysine 970 in the kinase domain represents the residue important for JAK2 activation.

### K63-linked poly-ubiquitination on K970 of JAK2 kinase domain is promoted by Cbl

**Figure 8:**
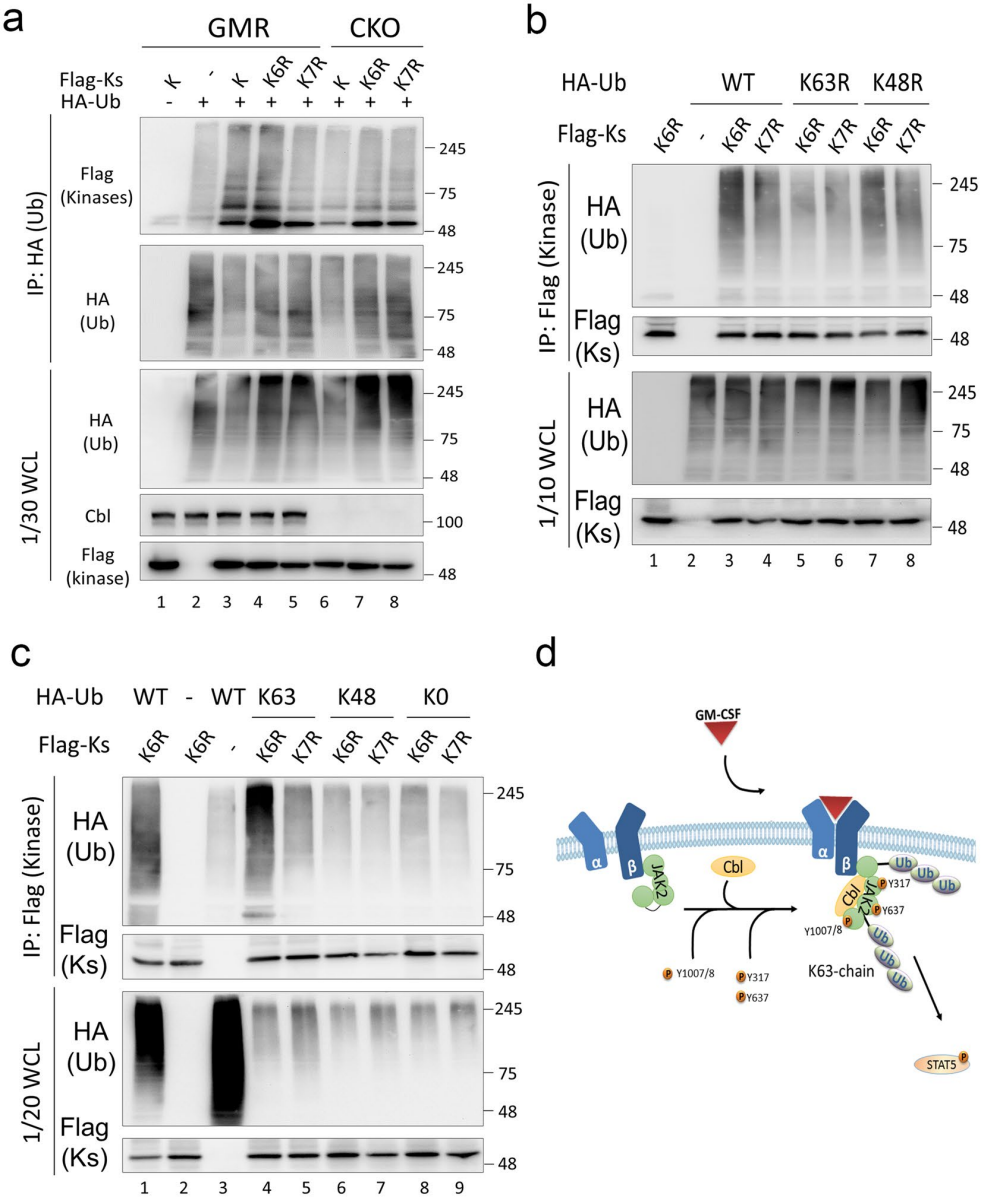
K63-linked poly-ubiquitination of JAK2 K970 is promoted by Cbl. (**a**) The ubiquitination of JAK2 K970 is decreased in absence of Cbl. Flag-tagged JAK2 kinase domain mutants (as described in Fig. 7c) and HA-tagged ubiquitin were co-expressed in GMR cells and CKO cells as described. WCLs were prepared and analysed as described in Fig. 1. (**b**) The ubiquitin K63R substitution greatly reduces the ubiquitination ability of JAK2 K970. JKO cells were co-transfected with Flag-tagged JAK2 kinase domain mutants (K6R and K7R) and HA-tagged WT, K63R, or K48R ubiquitin mutants. Transfected cells were treated with MG132 (10 μM) for 4 h before harvest. WCLs were prepared and analysed as described in Fig. 1c. (**c**) The K63-only mutant partially rescues ubiquitination on JAK2 K970. JKO cells were co-transfected with Flag-tagged JAK2 kinase mutants (K6R or K7R) and HA-tagged WT or K63-only (K63), K48-only (K48), or the no-Lys (K0) mutant. Cells were processed and analysed as described above. (**d**) A schematic model for Cbl-mediated JAK2 K63-linked poly-ubiquitination and activation upon GM-CSF stimulation based on research data presented in this manuscript.

To investigate whether ubiquitination on JAK2 K970 is Cbl dependent, HA-tagged ubiquitin and Flag-tagged JAK2 kinase domain-only mutants (K, K6R and K7R) were co-expressed in CKO or parental GMR cells. Following IP of HA-ubiquitin, the ubiquitinated smearing was detectable in the K6R mutant which preserved lysine 970 as well as the wild type kinase domain (K) in GMR cells, but this smearing was decreased in CKO cells (Fig.8a, compare lanes 3 and 4 to 6 and 7). Moreover, this ubiquitination was also diminished when the kinase domain incorporated a further mutated lysine 970 (K7R) in GMR cells (comparison of lanes 5 to 3 and 4), indicating that ubiquitination of lysine 970 is regulated by Cbl. The K48R or K63R mutant ubiquitination sites were also co-transfected with kinase domain mutants (K6R or K7R) to repeat the experiment. As shown in Fig. 8b and as previously seen in anti-Flag IPs, the WT and K48R ubiquitins yielded strongly positive signals of ubiquitination of the K6R JAK2 kinase domain (lanes 3 and 7), and lesser ubiquitination of K7R mutants (lanes 4 and 8). However, the K63R-ubiquitin mutant gave equivalent background signals for both the K6R and K7R JAK2 kinase domain mutants (comparison of lanes 5 and 6). Moreover, when we co-expressed Flag-tagged JAK2 kinase domain mutants (K6R or K7R) with either K48-, K63-only or no-lysine (K0) ubiquitin mutants, only the K63-only ubiquitin gave a strong signal, indicative of ubiquitination of the K6R substitution mutant, but much lesser signal in the K7R mutant (Fig.8c, compare lanes 4 to 6). Neither K48- nor K0 presented any significant ubiquitination signal of JAK2 kinase domains (lanes 6 to 9). Data from Fig. 8c is a mirror condition to that of Fig. 8b, and both suggest that lysine 970 on JAK2 is preferentially poly-ubiquitinated *via* K63-linkage.

## Discussion

In this study, we provided evidence suggesting that Cbl can positively control GM-CSF-induced JAK2 activation and signalling. Although many experiments suggest likely through direct interaction, further studies are required in order to determine whether the interaction between these two proteins, Cbl and JAK2, is indeed direct. By both gain-of-function and loss-of-function approaches, we were able to demonstrate that Cbl mediates GM-CSF-induced JAK2 poly-ubiquitination, which is predominantly K63-conjugated. Furthermore, as shown by Western blotting and a reporter gene assay, Cbl promotes GM-CSF-induced JAK2 activation and signaling. This function of Cbl is, at least in part, due to interaction with and ubiquitination of JAK2. A detailed analysis of the association between Cbl and JAK2 indicated that Cbl preferentially bind to and ubiquitinate both the kinase domain and the FERM-SH2 (JN) domain of JAK2, *via* the TKB domain of Cbl. Through point mutation scanning analyses and functional assays, our results suggested that ubiquitination at K970 in JAK2 plays an important role in promoting the full phosphorylation and activation of JAK2.

How K970 ubiquitination modulates the maximum level of JAK2 activation remains to be discerned. According to sequence and structure comparisons of the JAK protein family, K970 is non-conserved; the corresponding residues in JAK1 and JAK3 are both arginine (R), and a glutamine (Q) is found at this position in TYK2 (Supplementary Fig. S1). It is therefore clear that ubiquitination at K970 only occurs in JAK2. The crystal structure of the murine JAK2 kinase domain (PDB entry 4GL9)^38^ reveals that K970 is located near the C-lobe of the kinase domain and is exposed on the exterior surface of the molecule. Presumably, this surface localization indicates that K970 is highly accessible for post translational modification, e.g. ubiquitination. Since the loss of K970 ubiquitination not only affects phosphorylation of the entire kinase domain, but also the phosphorylation of Y317 in the FERM domain and Y637 in the pseudo-kinase domain, it appears that K970 ubiquitination is required for certain conformational changes to occur in the kinase domain that further affect domain-domain interactions during phosphorylation.

JAK2 ubiquitination has also been reported in leukemia cells treated with a small molecule de-ubiquitinase inhibitor, WP1130^39^. Consistent with the observation that the ubiquitin that accumulated as a result of de-ubiquitinase inhibition by WP1130 was predominantly K63-conjugated, our data also revealed that the majority of poly-ubiquitination on JAK2 in the hematopoietic cell line TF1 was also K63-conjugated. There was, however, a significant discrepancy between these two studies concerning the consequences of ubiquitination; in one study, JAK2 translocated to the perinuclear aggresome and decreased signaling, whereas in our observations JAK2 was highly phosphorylated and fully activated. We postulate that this could be due to either excessive accumulation of too many poly-ubiquitinated JAK2 proteins by the de-ubiquitinase inhibitor, or an off-target effect in addition to the deubiquitinase inhibition in the presence of WP1130. This would direct ubiquitinated JAK2 proteins to a completely different cellular fate.

In previous studies, 15% of patients with juvenile myelomonocytic leukemia (JMML), a disease that is characterized by GM-CSF hypersensitivity and resistance to chemotherapy, were identified containing somatic mutations in Cbl^30, 40, 41^. Bunda and colleagues have demonstrated that WT Cbl plays a regulatory role in controlling GM-CSF-mediated survival and growth responses^34^, and have proposed two pathways to account for the phenotypes of these Cbl mutant cells. The first proposal is that mutant Cbl increases cell proliferation signals by stabilizing labile phosphorylated Src, which in turn increases the levels of phosphorylation of mutant Cbl and GMRβ proteins and thereby results in activation of downstream Ras/MAPK/ERK signaling^32^. The second suggestion is that mutant Cbl promotes survival by stabilizing another Src family kinase, Lyn. An increase in Lyn phosphorylation promotes phosphorylation of Cbl and subsequently enhances phosphorylation of the p85 subunit of PI3K and thereby amplifies Akt signaling *via* the adaptor function of Cbl^34^. Here we report an additional function of Cbl, whereby it directly promotes K63-conjugated poly-ubiquitination and full phosphorylation of JAK2 during GM-CSF stimulation. This discovery emphasizes that we need to have a more detailed understanding of the functions of WT Cbl, as well as oncogenic Cbl, in order to be able to reconcile recent findings concerning these proteins. The mechanisms underlying the effects of oncogenic Cbl mutants on the association, ubiquitination, and activation of JAK2 warrants further investigation.

Although ample evidence shows that Cbl functions as a negative regulator of various activated receptor tyrosine kinases by mediating their poly-ubiquitination and degradation, as a multi-domain adaptor protein, Cbl also acts as a positive regulator in integrin signaling in macrophages^42^ and in the bone resorption activity of osteoclasts^43^. Recently Stanley and co-workers presented evidence that a Cbl-dependent pathway is required for CSF-1R ubiquitination, which permits full receptor tyrosine phosphorylation in activated macrophages^44^, despite the fact that several groups have reported the process of Cbl-mediated CSF-1R endocytosis and degradation in macrophages *via* direct^45^ or indirect mechanisms^46^. The Cbl-mediated CSF-1R ubiquitination likely causes conformational changes in the kinase domain that allows for full phosphorylation of the receptor tyrosine kinase. Studies on the NF-kB pathway have also revealed a similar mechanism, whereby K63-linked poly-ubiquitination of TAK1 facilitates protein kinase dimerisation and activation, which can then, in turn, phosphorylate and activate IkB kinase^47^. Therefore, the mechanism we now report in JAK2 activation, whereby K63-conjugated poly-ubiquitination functions as a conformational switch, which allows the JAK2 tyrosine kinase to be fully phosphorylated and maximally activated, may represent a more general mode of action that applies to many other kinases.

Although endogenous Cbl maximally interacts and ubiquitinates JAK2 when cells were stimulated by GM-CSF, Cbl can interact with the kinase domain of JAK2, which was expressed as a cytosolic protein. Therefore, we anticipate that Cbl can ubiquitinate both receptor-bound and cytosolic forms of JAK2, and that this does not need to occur in a receptor-dependent manner. This notion is also consistent with our JAK2 *in vitro* ubiquitination reaction by Cbl where GM-CSF receptor was not required, although the presence of receptor and GM-CSF stimulation can significantly enhance the interaction and ubiquitination of JAK2 by Cbl. We anticipate that this Cbl action is important for all JAK2-using cytokine receptor signaling. However, due to the complicated interaction networks between Cbl and receptors, signal adaptor proteins and Src-family tyrosine kinases, the final influence of Cbl on each cytokine receptor signaling would be expected to be receptor-specific and cell-type-specific.

## Methods

### Plasmids

pcDNA3-GMRα and pGMRβ-clip are pcDNA3 vector-based expression systems encoding GMRα and GMRβ-clip proteins, respectively, as previously described^35^. pGMRβ-clip was constructed by inserting the PCR-amplified clip coding region from pCLIP vector (New England BioLabs,Ipswich, MA, USA) into the C terminus of the GMRβ gene in pCDNA3-GMRβ. The Cbl cDNA was kindly provided by Dr. Wallace Y. Langdon (School of Pathology and Laboratory Medicine, University of Western Australia, Australia), and sub-cloned into the pcDNA6 vector. The Cbl C381A mutant was generated using the QuikChange Lightning Multi Site-Directed Mutagenesis Kit (Agilent Technologies, Santa Clara, CA, USA). The construction of plasmids encoding the sub-domains of Cbl was performed according to a previous publication^48^. The sequences of all the primers used for Cbl construction are listed in Table S1. Plasmids encoding HA-tagged ubiquitins (WT, K63R, K48R, K0, K63, and K48) were kindly provided by Dr. Yi-Ling Lin (Institute of Biomedical Sciences, Academia Sinica, Taiwan) and Dr. Li-Chung Hsu (Institute of Molecular Medicine, College of Medicine, National Taiwan University). Flag-JAK2 and its domains were generous gifts from Dr. Hengst Ludger (Medical Biochemistry Division, University of Innsbruck, Austria). Plasmids encoding GST-JAK2 and its sub-domains were generously provided by Dr. Lily Jun-shen Huang (Department of Cell Biology, University of Texas SouthWestern Medical Center, USA). JAK2 Lys-to-Arg (KR) mutants were generated using the Multi Site-Directed Mutagenesis Kit (Agilent Technologies); the sequences of the primers used are listed in Table S2. The pGL4-CISH reporter plasmid was a kind gift from Dr. Charles V. Clevenger (Department of Pathology, Virginia Commonwealth University, USA). The STAT5-binding site mutation in the CISH (pCISHmut) reporter gene was generated using a site-directed mutagenesis kit with primers whose sequences are listed in Table S3. A human Cbl-specific target sequence (GTATTTCTCCATTACCTAGG) and a human JAK2-specific target sequence (TTATCTGACCTTTCCATCTGG) were designed (http://crispr.mit.edu) for CRISPR/Cas9 knockout experiments and then cloned into the pZGB-RGN3 vector to yield pZG22C02-Cbl or pZG22C03-JAK2, respectively, and the plasmids were purchased from Zgenebio Biotech, Inc., (Taipei, Taiwan).

### Cell lines

293 T cells were maintained in Dulbecco’s modified Eagle’s medium (DMEM) (Corning Inc., Corning, NY, USA) containing 10% fetal bovine serum (FBS) (Hyclone, South Logan, UT, USA). HeLa cells stably expressing both GMRα and GMRβ-clip (GMR) were established by co-transfecting pcDNA3-GMRα and pcDNA3-GMRβ-clip plasmids, and transfected cells were selected by culturing in the presence of G418 (200 μg/mL). Cbl knockout (CKO) or JAK2 knockout (JKO) cells were generated by transfecting GMR cells with either pZG22C02-Cbl or pZG22C03-JAK2, followed by sorting of GFP-positive or mCherry-positive cells, respectively, by flow cytometry. HeLa-derived cell lines were maintained in the same medium as 293 T cells. TF1 cells were cultured in RPMI 1640 medium (Corning Inc.) containing 10% FBS and GM-CSF (1 ng/mL). CKO TF1 stable clones were established by transfecting TF1 cells with pZG22C02-Cbl, followed by sorting of GFP-positive cells by flow cytometry. CKO TF1 cells were maintained in the same medium as that used for parental TF1 cells.

### Antibodies

The following antibodies were used in this study: anti-GMRβ (1C1) and anti-STAT5(C-17) from Santa Cruz Biotechnology (Santa Cruz, CA, USA); anti-JAK2 (D2E12), anti-pJAK2 (Y1007/1008, #3771), anti-pSTAT5 (Y694, #9351), anti-Cbl (#8447), anti-ubiquitin (P4D1,#3936), and anti-pY (#9411) from Cell Signaling Technology (Beverly, MA, USA); anti-HA (GTX628489) from Genetex (Hsinchu, Taiwan); anti-Flag (M2) from Sigma-Aldrich (Saint Louis, MO, USA); anti-JAK2 (04-001) and anti-GST (06-322) from Merck Millipore (Billerica, MA, USA). Anti-pJAK2Y317 and pJAK2Y637 antibodies were kindly provided by Dr. Martin G. Myers, Jr. (Department of Internal Medicine, University of Michigan Medical School).

### Other reagents and chemicals

Anti-Flag M2 affinity gels and EZview Red HA-Agarose beads were purchased from Sigma-Aldrich. Tandem Ubiquitin Binding Entity (TUBE) −1 and −2 agarose beads were purchased from LifeSensors (Malvern, PA, USA). G418, *N*-ethylmaleimide and iodoacetate were purchased from Sigma-Aldrich; GM-CSF was purchased from Gentaur (Kampenhout, Belgium).

### Immunoprecipitation (IP) and Western blotting

Cells were lysed in either 1% Triton X-100 or 1% NP-40 lysis buffer, supplemented with protease and phosphatase inhibitors, and whole cell lysates (WCLs) were IPed and analysed by Western blotting using the indicated antibodies. In order to detect protein ubiquitination, cells were treated with MG132 (10 μM) for 4 h before harvest, and *N*-ethylmaleimide (20 mM) and iodoacetate (5 mM) were used in the lysis buffer.

### Duolink® *in situ* proximity ligation assay

Endogenous protein-protein interactions were detected using a Duolink® PLA kit (Sigma-Aldrich) according to the manufacturer’s instructions. Briefly, after serum starvation, TF1 cells were treated with or without GM-CSF (1 ng/mL) for the indicated times and were then attached to glass slides by cytospinning. The slides were fixed in 4% paraformaldehyde for 15 min at room temperature, permeabilized with 0.1% Triton X-100 in PBS, and blocked with Duolink® blocking buffer for 30 min at 37 °C. The rabbit anti-human Cbl antibody and either a mouse anti-human GMRβ or a mouse anti-human JAK2 antibody in Duolink® dilution buffer were incubated with the slides for 16–18 h at 4 °C. After washing three times, Duolink® PLA immune-probes targeted against the two different primary antibodies (rabbit and mouse) were added for 1 h at 37 °C. If the antibodies are in close proximity (<40 nm), these probes can hybridize to form a circular DNA molecule. The circular DNA molecules were then covalently ligated using the Duolink® ligation solution for 30 min at 37 °C. The covalent circular DNA was then amplified by rolling circle amplification using the Duolink® polymerase for 100 min at 37 °C. Amplified DNA was then detected by hybridization of Duolink® detection probes and signals were detected by confocal microscopy using a Zeiss LSM780 and ImageXpress Micro Confocal High-Content Imaging System.

### *In vitro* ubiquitination assay

Flag-tagged JAK2 and HA-tagged Cbl proteins were IPed from Cbl knockout 293 T cells using an anti-Flag M2 affinity gel or EZview Red HA-Agarose beads, and eluted with 0.1 M glycine-HCl pH 3.5. Eluted proteins were incubated with recombinant E1, E2 (Ubc13), and biotin-labelled ubiquitin in ubiquitination buffer (50 mM Tris-HCl pH 8.0, 5 mM MgCl_2_, 1 mM DTT, 2 mM ATP, and 2 mM ubiquitin aldehyde) at 37 °C for 4 h. Biotin-labelled ubiquitin and E1 were purchased from Enzo Life Sciences (Farmingdale, NY, USA); GST-Ubc13 was a kind gift from Dr. Sheau-Yann Shieh (Institute of Biomedical Sciences, Academia Sinica, Taiwan). After incubation, the reactions were analysed by Western blotting.

### Luciferase reporter activity

GMRαβ or CKO HeLa cells were seeded into 24-well plates for transfection. Reporter plasmids pGL4-CISH or pCISHmut (50 ng) were co-transfected with the *Renilla* luciferase reporter plasmid (0.5 ng) and/or other indicated plasmids. After transfection, cells were maintained in DMEM culture medium for 24 h, and then changed to fresh serum-free DMEM with or without 10 nM GM-CSF for an additional 24 h. Cells were then harvested and assayed for luciferase activity using Luc-Pair™ firefly luciferase HS assay kit (GeneCopeia, Germantown, MD, USA). Briefly, the cells were washed in PBS and lysed with Luc-Lysis II Buffer at room temperature for 15 min, and then analysed for firefly luciferase activity (FLuc assay working solution) and *Renilla* luciferase activity (RLuc assay working solution). Each assay was performed at least four times.

### Statistical analysis

Protein phosphorylation levels from Western blots were quantified using ImageJ software (version 1.63 f, National Institutes of Health, USA).

Data are expressed as the mean ± SEM from three or more independent experiments and analysed with either Student’s t-tests (for comparison between two groups) or one-way analysis of variance (ANOVA) using Microsoft Excel.

## Electronic supplementary material

supplementary information
