# Supplementary material for: Cbl-mediated K63-linked ubiquitination of JAK2 enhances JAK2 phosphorylation and signal transduction
Source: Sci Rep. 2017 Jul 4;7:4613. doi: 10.1038/s41598-017-04078-w (PMC5496907; doi:10.1038/s41598-017-04078-w)
Supplement: Supplementary file 1 — supplementary information [file 41598_2017_4078_MOESM1_ESM.pdf]

Cbl-mediated K63-linked ubiquitination of JAK2 enhances JAK2 phosphorylation and signal transduction

Chun-Shan Liu[1,2], Hsin-Fang Yang-Yen[3], Ching-Shu Suen[2], Ming-Jing Hwang[2], and Jeffrey Jong-Young Yen[1,2*]

[1]Graduate Institute of Life Sciences, National Defense Medical Center, Taipei, Taiwan, ROC; [2]Institute of Biomedical Sciences and [3]Institute of Molecular Biology, Academia Sinica; Taipei, Taiwan ROC;

***Correspondence** should be sent to Jeffrey J.Y. Yen, Institute of Biomedical Sciences, Academia Sinica, No. 128, Sec. 2, Yen-Jiou-Yuan Rd., Taipei 11529, Taiwan; e-mail: bmjyen@ibms.sinica.edu.tw; Phone: 886-2-26523077; FAX 886-2-26523081.

## Supplementary Methods

### Sequence analysis

Sequence alignment was produced using ClustalW Omega[1] and formatted with BOXSHADE, written by K. Hofmann and M. Baron. (http://www.ch.embnet.org/software/BOX_form.html).

## Supplementary tables

### Table S1: Primers used for Cbl construction

| | |
|---|---|
| Cbl-F1: | 5'- gcagatctcgccaccatggccggcaacgtgaagaagag-3' |
| Cbl-F2: | 5'- gcagatctcgccaccatgtgtaaaatatgtgctgaaaatgataaggatgtaaagattg-3' |
| Cbl-R1: | 5'- gcggatccgccagtcagatcaggattctgatttcg-3' |
| Cbl-R2: | 5'- gcggatcctagttggaatgtggagcccatctc-3' |
| Cbl-R3: | 5'- gcggatccggtagctacatgggcaggagaag -3' |
| Cbl C381A: | 5'-gagatgggctccacattccaactaGCtaaaatatgtgctgaaaatgataa-3' |

### Table S2: Primers used for generating JAK2 KR mutants

| | |
|---|---|
| K191R: | 5'-gacatgatgagaatagctaGggagaaagaccagactc-3' |
| K193R: | 5'-catgatgagaatagctaaggagaGagaccagactcc-3' |

| | |
|---|---|
| K212R: | 5'-agctacaagacattcttaccaaGgtgcgttcgagc-3' |
| K217R: | 5'-aaagtgcgttcgagcgaGgatccaagactatcaca-3' |
| K244R: | 5'-tcattcagcaattcagtcaatgtaGagccactgccagg-3' |
| K762R: | 5'-gagtgctctggattctcaaagaaGgctgcagttctatg-3' |
| K769R: | 5'-agaaagctgcagttctatgaagataGgcatcagcttcct-3' |
| K850R: | 5'-ttgaagagagacacttgaGgtttctacagcagcttgg-3' |
| K882R: | 5'-ggcgaggtggtcgctgtgaGgaaactccagcac-3' |
| K883R: | 5'-ggcgaggtggtcgctgtgaagaGactccagcac-3' |
| K912,914R: | 5'-tgcagcatgacaacatcgtcaGgtacaGgggagtgtgct-3' |
| K970R: | 5'-gggcatggaatatcttggtacaaGaaggtatatccacagg-3' |

**Table S3: Primers used for generating CISH mutant**

| | |
|---|---|
| Mut1: | 5'-cttgacctcagtggacccactcatttAtACaactgtcctctgca-3' |
| Mut2: | 5'-cccgccccgttttcctggaaagttTAtACaaatctgtcaaagtatttcctttc-3' |
| Mut3: | 5'-cgctcagcccgcggttTtaACaagacgctgcttccg-3' |
| Mut4: | 5'-gttctaggaagacgctgcttTAgACaagggctggaacgcgg-3' |

## Supplementary Figures

### Figure S1

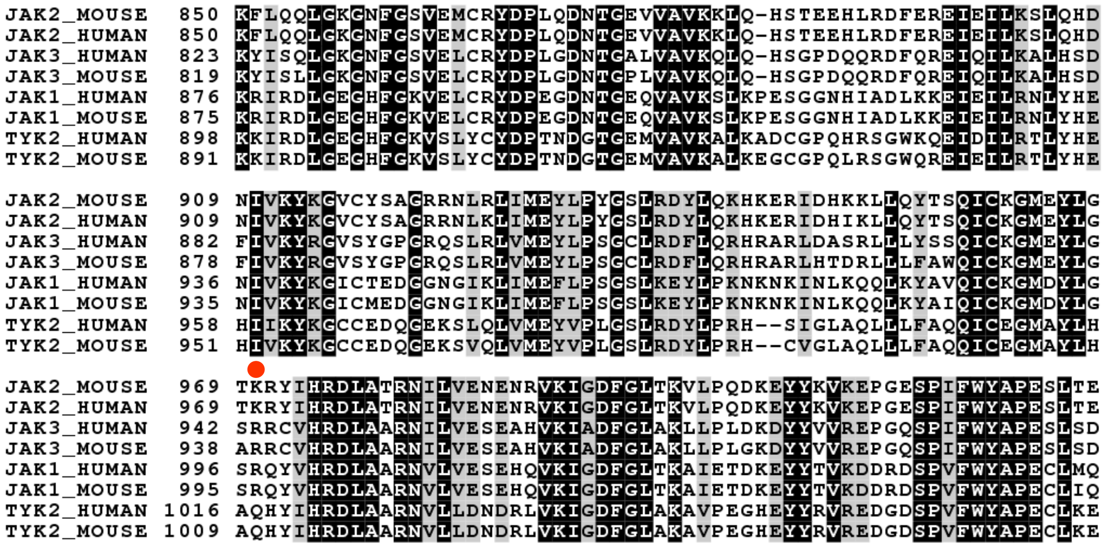


**Figure S1. Multiple sequence alignment of the kinase domain of human and mouse JAK family.** Sequence identical regions are shaded in black while similar regions are in grey background. Numbers to the left of sequences denote the amino acid positions. The red-cycle indicates the location of JAK2 K970.

### References for supplementary information

1 Sievers, F. *et al.* Fast, scalable generation of high-quality protein multiple sequence alignments using Clustal Omega. *Mol Syst Biol* **7**, ARTN 539 doi:10.1038/msb.2011.75 (2011).
